# Variation in fluoroquinolone pharmacodynamic parameter values among isolates of two bacterial pathogens of bovine respiratory disease

**DOI:** 10.1038/s41598-018-28602-8

**Published:** 2018-07-12

**Authors:** Xuesong Wen, Ronette Gehring, Jim E. Riviere, Brian V. Lubbers, Tara Nath Gaire, Bre’Anna Wyche, Breanna Fox, Victoria Quichocho, Victoriya V. Volkova

**Affiliations:** 1 Department of Anatomy and Physiology, Institute of Computational Comparative Medicine, College of Veterinary Medicine, Kansas State University, 1800 Denison avenue, Manhattan, KS 66506 USA; 2 Department of Diagnostic Medicine/Pathobiology, College of Veterinary Medicine, Kansas State University, 1800 Denison avenue, Manhattan, KS 66506 USA

## Abstract

To design an antimicrobial treatment regimen for a bacterial disease, data on the drug pharmacodynamics (PD) against selected drug-susceptible strains of the pathogen are used. The regimen is applied across such strains in the field, assuming the PD parameter values remain the same. We used time-kill experiments and PD modeling to investigate the fluoroquinolone enrofloxacin PD against different isolates of two bovine respiratory disease pathogens: four *Mannheimia haemolytica* and three *Pasteurella multocida* isolates. The models were fitted as mixed-effects non-linear regression; the fixed-effects PD parameter values were estimated after accounting for random variation among experimental replicates. There was both inter- and intra- bacterial species variability in the PD parameters Hill-coefficient and *E*_max_ (maximal decline of bacterial growth rate), with more variable PD responses among *M. haemolytica* than among *P. multocida* isolates. Moreover, the Hill-coefficient was correlated to the isolate’s maximal population growth rate in the absence of antimicrobial exposure (a.k.a. specific growth rate; Spearman’s *ρ* = 0.98, *p*-value = 0.003, *n* = 6 isolates excluding one outlier). Thus, the strain’s properties such as growth potential may impact its PD responses. This variability can have clinical implications. Modifying the treatment regimen depending on phenotypic properties of the pathogen strain causing disease may be a precision medicine approach.

## Introduction

Effective antimicrobial treatment regimens for bacterial diseases are critical for achieving therapeutic success. Treatment regimens (*i.e*. the drug dose, dosing interval, and duration) are typically designed considering the antimicrobial pharmacokinetics (PK) and the antimicrobial pharmacodynamics (PD) against the pathogen causing the disease^1^. The pharmacodynamics can be described by multi-parametric mathematical models, such as the maximum effect (*E*_max_) model based on the Hill function^2,3^. Such models capture the relationship between the antimicrobial drug concentration and its effect on the growth rate of the exposed pathogen population^2,3^. These models can then be linked with those of the *in vivo* drug PK in the host to predict microbiological efficacy of the treatment regimen^4–6^. The regimen may be chosen based on the antimicrobial’s PK and PD characteristics for the bacterial pathogens most often involved in the disease etiology for those diseases often caused by multiple pathogens. The antimicrobial’s PD against each specific pathogen is described using *in vitro, ex vivo*, or *in vivo* studies conducted with selected drug-susceptible strains of the pathogen; the strains are judged as susceptible based on the antimicrobial’s minimum inhibitory concentration, MIC, for the strains. Then, the chosen regimen is applied across the infections by drug-susceptible strains in the field. It is assumed that values of the other PD parameters (*e.g*. the Hill-coefficient and *E*_max_) remain the same across the strains classified as drug-susceptible based on the MIC, are independent of other strain properties, and do not to change during the treatment.

We consider bovine respiratory disease (BRD) as an example of a multi-pathogen disease, where *Mannheimia haemolytica* and *Pasteurella multocida* are bacterial pathogens most often involved in the etiology^7,8^. A fluoroquinolone, enrofloxacin, is approved by the U.S. Food and Drug Administration with a single regimen for the treatment of BRD associated with either of these two respiratory pathogens^9^. Differences in the PD of the fluoroquinolone marbofloxacin between the drug-susceptible based on the MIC *M. haemolytica* and *P. multocida* have been reported in recent studies^10,11^. Specifically, the value of the PD parameter Hill-coefficient (*H*, which reflects how sensitive the bacterial population growth rate is to an increase in the antimicrobial concentration during the initial dynamic phase of the population-antimicrobial interactions) was observed to be higher for *M. haemolytica* than for *P. multocida*^10^. The value of the parameter reflecting the maximum effect on the bacterial population growth rate (*E*_max_) was observed to be lower for *M. haemolytica* than for *P. multocida*^10^. However, only one isolate of each of the two pathogens was investigated in that study. Other authors considered as a PD outcome the absolute difference between the initial bacterial population size and the persister subpopulation remaining after 24 hours of the antimicrobial exposure. That outcome was also different between *M. haemolytica* and *P. multocida* exposed to the fluoroquinolone marbofloxacin^11^, while intra-species differences among strains for this outcome were limited^11^. Differences in the PD responses inter- and intra- bacterial species can impact on which treatment regimen by the antimicrobial can achieve therapeutic success.

The goal of the present study was to investigate whether the reported differences in fluoroquinolone PD between *M. haemolytica* and *P. multocida* are solely a function of the bacterial species, or possibly a function of individual bacterial strains. We used the strains that would not be classified as fluoroquinolone-resistant based on the antimicrobial’s MIC. This is clinically important, for if the antimicrobial’s PD profile varies among the drug-susceptible strains within a specific species, than individualized treatment regimens (which may not be reflected in the current drug label) may need to be considered for effective therapy. This targeted hypothesis could be investigated by comparing the PD parameter values intra- and inter- the two bacterial species during the initial dynamics of the bacterial population growth/decline when exposed to various concentrations of the antimicrobial. This basic-science rooted approach considers the initial dynamics as its own outcome (*i.e*., apart from the size of the persister bacterial subpopulation after a 20 + hour exposure to the antimicrobial)^3,6,12–15^. We compared the initial *in vitro* PD of the fluoroquinolone enrofloxacin for several isolates of *M. haemolytica* and *P. multocida*, by comparing the parameter values of the *E*_max_ model for the isolates.

## Results

### Intra- and inter- bacterial species variation in fluoroquinolone pharmacodynamics

**Figure 1:**
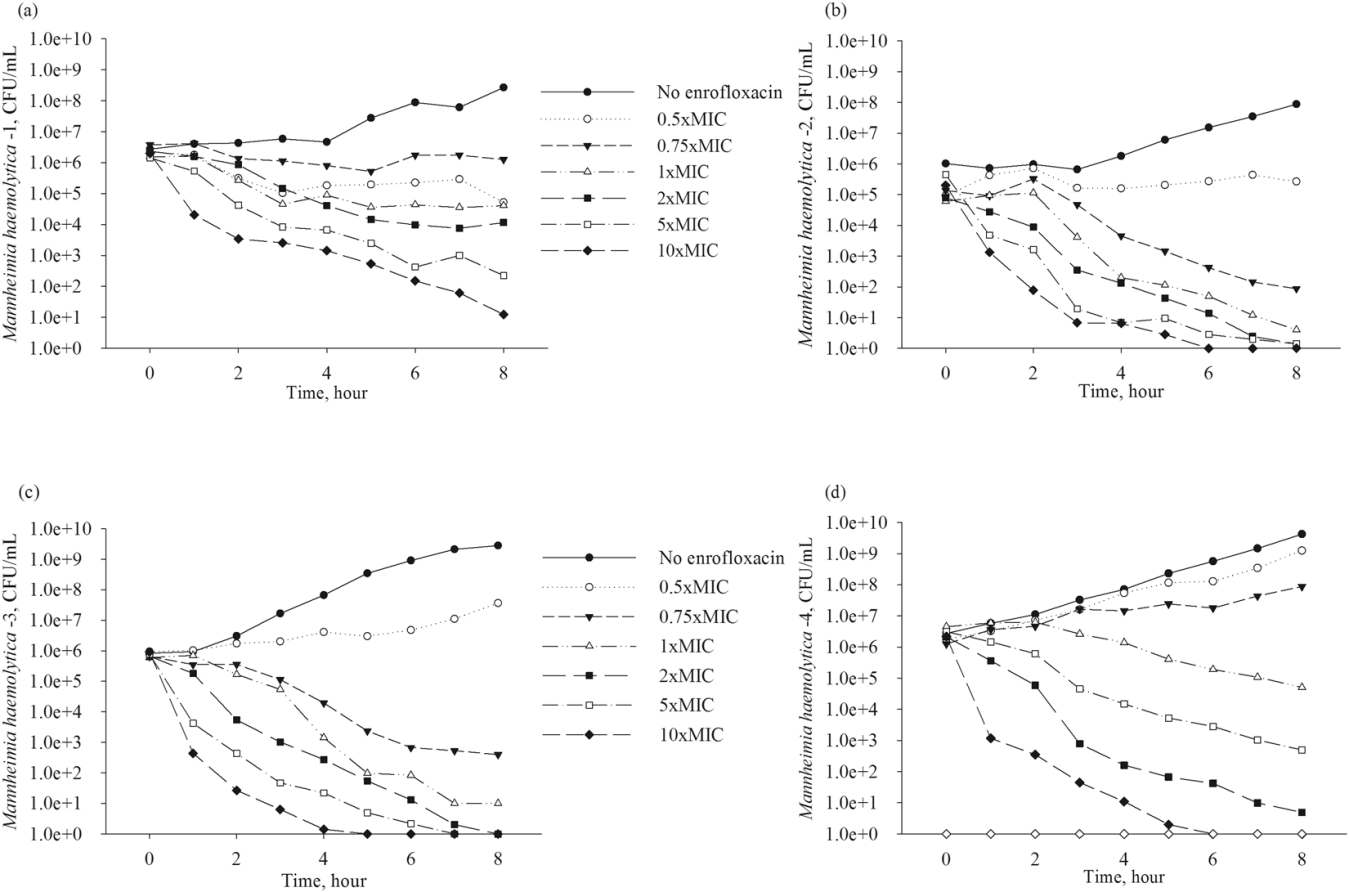
Data from time-kill experiments with the fluoroquinolone enrofloxacin for four isolates of *Mannheimia haemolytica* from bovine respiratory disease cases. For each isolate and enrofloxacin concentration combination, the average result of at least two replicates of the experiment is plotted. (The isolate number corresponds to that in Table 1).

**Figure 2:**
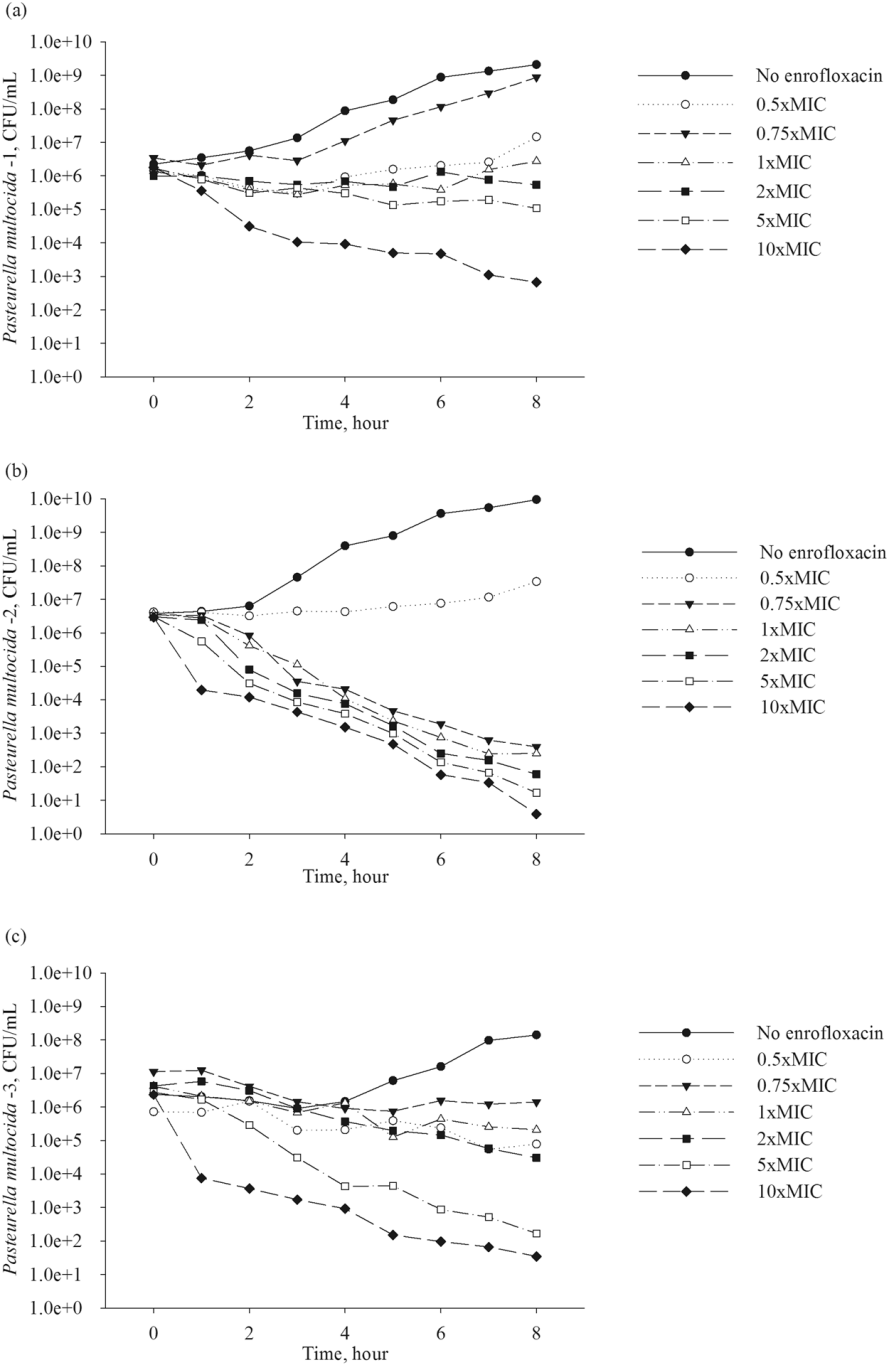
Data from time-kill experiments with the fluoroquinolone enrofloxacin for three isolates of *Pasteurella multocida* from bovine respiratory disease cases. For each isolate and enrofloxacin concentration combination, the average result of at least two replicates of the experiment is plotted. (The isolate number corresponds to that in Table 1).

Time-kill experiments were conducted with enrofloxacin for each of four *M. haemolytica* isolates and three *P. multocida* isolates (no further isolates of *P. multocida* were studied because a relatively low variation in enrofloxacin PD parameter values was observed among the isolates of this pathogen, as is detailed below). The data from the time-kill experiments for *M. haemolytica* isolates are plotted in Fig. 1a–d and for *P. multocida* isolates in Fig. 2a–c. (The data for the *P*. *multocida* isolate - 1 have been also presented earlier^12^). For each isolate, the *E*_max_ model (Eq. 1) was fitted to the time-kill data for the initial dynamics of the bacterial population growth/decline when exposed to the antimicrobial (the linear phase of the bacterial population increase or decline). The model (Eq. 1) was fitted as a mixed-effect non-linear regression model, with the time-kill experiment date added as the random effect to account for random variability in the experimental conditions between the days (*i.e*., between the experiment replicates).

1 $$E(C)={E}_{0}-\frac{{E}_{\max }\times {C}^{H}}{E{{C}_{50}}^{H}+{C}^{H}}$$

where:

$E(C)$- bacterial population growth rate when exposed to the antimicrobial concentration *C* (log_10_(colony forming units (CFU/mL)))

*C*- antimicrobial concentration (μg/mL)

${E}_{0}$- bacterial population growth rate in the absence of antimicrobial exposure (log_10_(CFU/mL))

$E{C}_{50}$- concentration of the antimicrobial that achieves a 50% of the maximal decline in the bacterial population growth rate (μg/mL)

${E}_{\max }$- maximal decline of the bacterial population growth rate when exposed to the antimicrobial (dimensionless)

*H*- Hill-coefficient reflecting steepness of the relationship between an increase in the antimicrobial concentration and an increase in the decline of the bacterial population growth rate (dimensionless).

The isolates of *M. haemolytica* varied in the sensitivity of the population growth/decline rate to an increase in enrofloxacin concentration. For two of the *M. haemolytica* isolates, the sensitivity was relatively low, with a relatively small increase in the population decline with an increase in enrofloxacin concentration (Fig. 1a,b). For the other two *M. haemolytica* isolates, the sensitivity was high, with a large increase in the population decline with an increase in enrofloxacin concentration (Fig. 1c,d). The pharmacodynamics were more variable among *M. haemolytica* isolates than among *P. multocida* isolates (Fig. 1 *vs*. Fig. 2). Also, the PD for *P. multocida* isolates were more similar to the first group of *M. haemolytica* isolates with a relatively low sensitivity of the population growth/decline rate to an increase in enrofloxacin concentration (Figs 1a,b and 2).

**Figure 3:**
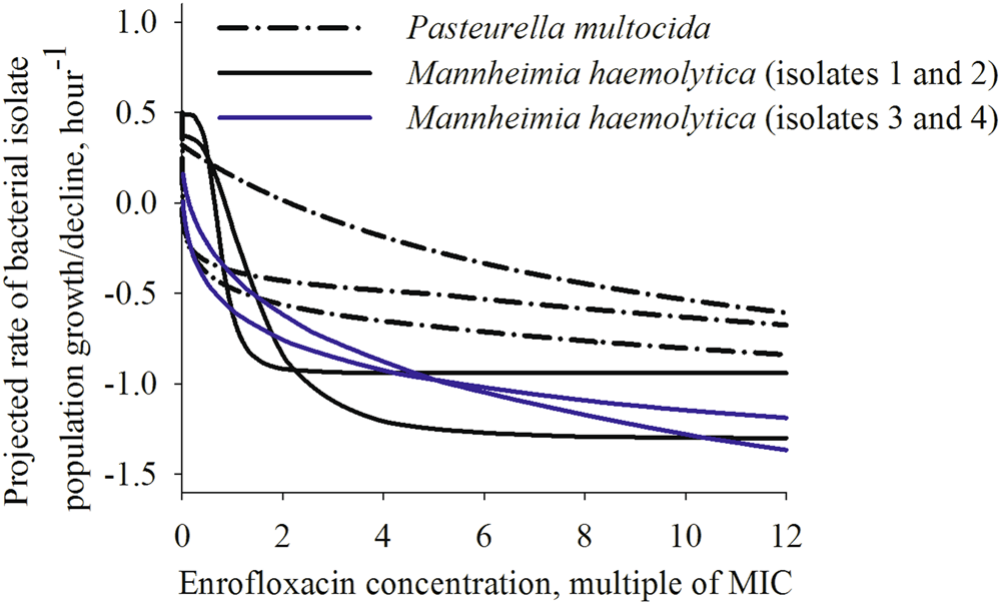
Projected rates of population growth/decline of three isolates of *Pasteurella multocida* and four isolates of *Mannheimia haemolytica* when exposed to different concentrations of the fluoroquinolone enrofloxacin. (The isolate number corresponds to that in Table 1).

The parameter values of the best-fit *E*_max_ model for enrofloxacin PD for each of the seven isolates are given in Table 1. Using these estimated *E*_max_ model parameter values, the population growth/decline of each isolate at different enrofloxacin concentrations was projected. These generalized projections were plotted together and this further visualized that the PD responses were more variable among *M. haemolytica* than among *P. multocida* isolates (Fig. 3). There were also apparent inter-species differences, beyond the intra-species between-isolate variation; the maximal population decline tended to be greater for *M. haemolytica* than for *P. multocida* (Fig. 3).

**Table 1:** Parameter values of the sigmoid *E*_max_ pharmacodynamic (PD) models for the fluoroquinolone enrofloxacin for four isolates of *Mannheimia haemolytica* and three isolates of *Pasteurella multocida* from bovine respiratory disease cases.

Bacterial species-isolate	MIC (µg/mL) of enrofloxacin	PD parameter	Parameter value estimate (95% CI of the estimate)	CV (%) of the estimate
*M. haemolytica*-1	0.01	*E*_0_, log(CFU/mL) × hour^−1^	0.25 (0.06, 0.43)	34
		*E*_max_	1.46 (0.87, 2.05)	19
		*EC*_50_, µg/mL	0.03 (0.01, 0.05)	35
		*H*	0.43 (0.12, 0.74)	33
*M. haemolytica*-2	0.05	*E*_0_, log(CFU/mL) × hour^−1^	0.26 (0.20, 0.32)	11
		*E*_max_	3.45 (1.68, 5.23)	24
		*EC*_50_, µg/mL	0.75 (0.24, 1.26)	31
		*H*	0.53 (0.38, 0.68)	13
*M. haemolytica*-3	0.04	*E*_0_, log(CFU/mL) × hour^−1^	0.49 (0.46, 0.51)	2
		*E*_max_	1.43 (1.20, 1.67)	8
		*EC*_50_, µg/mL	0.03 (0.025, 0.03)	3
		*H*	4.21 (2.84, 5.59)	15
*M. haemolytica*-4	0.50	*E*_0_, log(CFU/mL) × hour^−1^	0.37 (0.29, 0.45)	10
		*E*_max_	1.68 (1.10, 2.25)	16
		*EC*_50_, µg/mL	0.69 (0.28, 1.11)	28
		*H*	2.58 (0.35, 4.81)	40
*P. multocida*-1	0.01	*E*_0_, log(CFU/mL) × hour^−1^	0.32 (0.26, 0.38)	9
		*E*_max_	1.64 (1.34, 1.93)	8
		*EC*_50_, µg/mL	0.11 (0.08, 0.13)	11
		*H*	0.97 (0.73, 1.22)	12
*P. multocida*-2	0.02	*E*_0_, log(CFU/mL) × hour^−1^	0.50 (0.47, 0.53)	3
		*E*_max_	2.53 (2.19, 2.87)	6
		*EC*_50_, µg/mL	0.14 (0.07, 0.20)	21
		*H*	0.21 (0.04, 0.37)	37
*P. multocida*-3	0.01	*E*_0_, log(CFU/mL) × hour^−1^	0.25 (0.12, 0.38)	25
		*E*_max_	1.74 (1.44, 2.03)	8
		*EC*_50_, µg/mL	0.25 (0.21, 0.28)	7
		*H*	0.19 (0.06, 0.32)	33

### Potential relationships between the bacterial isolate growth rate and fluoroquinolone pharmacodynamics against the isolate

**Figure 4:**
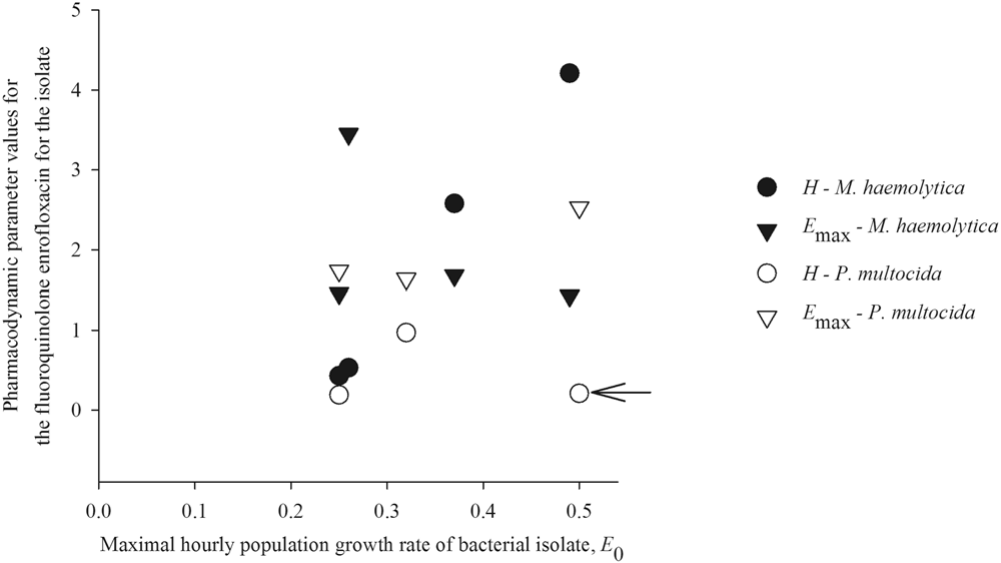
Relationships between values of individual parameters of the sigmoid *E*_max_ pharmacodynamic model for the fluoroquinolone enrofloxacin for an isolate of *Pasteurella multocida* (*n* = 3 studied) or *Mannheimia haemolytica* (*n* = 4 studied). Solid black symbols denote the data-points for *M*. *haemolytica* and white with black edge symbols the data-points for *P*. *multocida*. ${E}_{0}$- bacterial population growth rate in the absence of antimicrobial exposure. ${E}_{\max }$- maximal decline of the bacterial population growth rate when exposed to the antimicrobial. *H* – Hill-coefficient reflecting steepness of the relationship between an increase in the antimicrobial concentration and an increase in the decline of the bacterial population growth rate. The arrow identifies the outlier isolate in terms of the hypothesized relationship of *H* increasing with a higher ${E}_{0}$.

The results of the time-kill experiments and PD modeling suggested that the PD responses may depend on properties of the individual isolate within the pathogenic bacterial species. With the aim to identify the predictive properties, we analyzed relationships among the parameter values in the *E*_max_ model across the seven isolates studied. This analysis showed the isolate’s maximal population growth rate in the absence of antimicrobial exposure (a.k.a. specific growth rate) may be one of the properties impacting on the PD responses. With the exception of one outlier isolate, the *H* value increased with a higher maximal population growth rate *E*_0_ of the isolate across the *M. haemolytica* and *P. multocida* isolates studied (Fig. 4) (Spearman correlation coefficient 0.98, *p*-value = 0.003, *n* = 6 isolates). No systematic relationship between the *E*_0_ value and *E*_max_ value was observed (Fig. 4) (Spearman correlation coefficient *p*-value = 0.905, *n* = 7 isolates).

No systematic relationship was observed between the isolate’s *E*_0_ or PD responses and its susceptibility to enrofloxacin as measured by the MIC (*p*-value ≥0.150 for the Spearman correlation coefficient between the MIC and the value of either *E*_0_, *H*, *E*_max_ or *E*_50_; *n* = 7 isolates). However, the MIC value range was limited by design, because primarily isolates susceptible to enrofloxacin were included in the study (Table 1).

## Discussion

This study was aimed at investigated a targeted hypothesis whether differences reported for the fluoroquinolone PD against two bacterial species commonly involved in a multi-pathogen respiratory disease are solely a function of the species, or possibly also a function of individual bacterial strains intra-species. Bacterial isolates that would not be classified as fluoroquinolone-resistant based on the antimicrobial’s MIC were considered. Isolates of the respiratory pathogen *M. haemolytica* varied in their PD responses to different concentrations of the fluoroquinolone enrofloxacin; the time-kill experimental data are given in Fig. 1 and the generalized PD projections in Fig. 3. The estimated *E*_max_ model parameter values used for the projections are given in Table 1. Two of the *M. haemolytica* isolates (isolates 1 and 2 in Table 1 and Fig. 1a,b) demonstrated a sharply S-shaped curve of the relationship between an increase in the bacterial population decline with an increase in the drug concentration at <4x (isolate MIC), with no further increase in the population decline at the drug concentrations ≥4x (isolate MIC) (Fig. 3). This relationship was similar to that reported for *M. haemolytica* in an earlier study comparing PD of the fluoroquinolone marbofloxacin against *M. haemolytica* and *P. multocida*^10^. For these *M. haemolytica*, the *H* values were 0.43 and 0.53, while the *E*_max_ values were 1.46 and 3.45, respectively. The other two *M. haemolytica* isolates (isolates 3 and 4 in Table 1 and Fig. 1c,d) demonstrated a high sensitivity to an increase in the drug concentration, with a continuing increase in the bacterial population decline with an increase in the drug concentration even above 4x (isolate MIC) (Fig. 3). For these *M. haemolytica*, the *H* values were 4.21 and 2.58, while the *E*_max_ values were 1.43 and 1.68, respectively. Isolates of the respiratory pathogen *P. multocida* were less variable in the pharmacodynamic responses to different concentrations of the fluoroquinolone enrofloxacin. This can be seen in the results of the time-kill experiments in Fig. 2, and the generalized PD projections using the *E*_max_ model of the isolate population growth/decline dependency on the drug concentration in Fig. 3 (the model parameter value estimates used for the projections are given in Table 1). The magnitude of population decline of all *P. multocida* isolates continued to increase even at the drug concentrations above 4x (isolate MIC) (Fig. 3). The *E*_max_ value for *P. multocida* isolates ranged 1.64–2.53, while the *H* value showed a relatively greater variation with the range 0.19–0.97 (Table 1).

At the same time, an inter-species variability in the fluoroquinolone PD responses was observed for these two pathogens commonly involved in the etiology of one disease (BRD). The magnitude of the population decline of all four *M. haemolytica* isolates at enrofloxacin concentrations ≥4x (isolate MIC) was greater compared to all three *P. multocida* isolates (Fig. 3). These results agree with the earlier reports that investigated other isolates of these species and concluded inter-species differences in the fluoroquinolone PD for these respiratory pathogens^10,11^. It should be noted however that no significant difference in the maximal population decline (difference between the initial bacterial population size and the persister subpopulation remaining after 24 hours, CFU/mL), was observed between *M. haemolytica* and *P. multocida* isolates when exposed for 24 hours to the fluoroquinolone marbofloxacin in serum and other *ex vivo* body fluids^16^.

The results demonstrated a marked variation in the PD responses to the fluoroquinolone enrofloxacin among individual isolates of pathogenic bacterial species, *M. haemolytica* and *P. multocida* (Figs 1, 2 and 3). To investigate which isolate properties may influence the responses, we analyzed the relationships between values of the PD parameters for an isolate (Fig. 4, the parameter values are given in Table 1). This analysis suggested that the PD responses during the antimicrobial exposure may depend on such properties of the isolate as the maximal population growth rate during the exponential phase in the absence of the exposure (a.k.a. isolate’s specific growth rate). For six of the *M. haemolytica* and *P. multocida* isolates studied (after the exclusion of one outlier isolate), an isolate with a greater maximal population growth rate *E*_0_ in the absence of antimicrobial exposure experienced a steeper increase in the population decline with an increase in enrofloxacin concentration during the exposure, as reflected in a greater *H* value (Fig. 4). No systematic relationship was observed between *E*_0_ and the achieved maximal decline of the population growth rate during the exposure, *E*_max_ (Fig. 4).

Translation of the study findings to *in vivo* conditions will be complicated by that the BRD pathogen populations in the animal will be exposed to both enrofloxacin and its antimicrobially-active metabolites^17,18^. Thus, future studies could be designed to characterize the combined PD effects of the parent drug and its metabolites on the mono- or multi-pathogen populations. Also, the antimicrobial’s MIC for a given bacterial strain depends on the starting bacterial concentration for which the MIC is measured^15,19^. Values of the other PD parameters may be also changing depending on the bacterial concentration at the start and during the treatment. This may lead to yet unknown differences in the fluoroquinolone PD due to a different pathogen density between animals depending on the stage of the infectious disease (*e.g*., between therapeutically-treated animals with acute BRD *vs*. metaphylactically-treated animals in which the infection has not yet caused clinical BRD). To our knowledge, only data on the *M*. *haemolytica* density at the site of infection during acute BRD are available^20^. In this study, the starting bacterial density in the time-kill experiments was monitored to avoid the possible inoculum effect (Figs 1 and 2). Interested readers could pursue literature on investigations of potential role of the inoculum effect for fluoroquinolone treatment efficacy *in vivo* (see for example^10,21^).

Our study is limited to investigating the initial dynamic phase of the interactions between the antimicrobial and bacterial population; we consider this as a separate outcome from the size of the persister bacterial subpopulation remaining after 20+ hours of the antimicrobial exposure^3,6,12–15^. For the studied pathogens, our^12^ and others^11^ earlier data showed this initial dynamic phase is limited to the first 8 hours in the time-kill experiments.

Another limitation of this study is that field isolates of *P. multocida* and *M. haemolytica* (from samples submitted to the Kansas State Veterinary Diagnostic Laboratory) were used. The isolates were selected to provide temporal and geographical diversity, with no isolates originating from the same source premise (farm), and thus the isolates were assumed to be unrelated. However, the extent of circulation of individual *P. multocida* and *M. haemolytica* strains within a geographical area is not known. This imposes a risk that the studied isolates demonstrating similar PD responses and interpreted as unrelated isolates may have originated from a common ancestral strain.

In conclusion, the results of this *in vitro* study demonstrated a marked inter- and intra- bacterial species variation in the PD responses to the fluoroquinolone enrofloxacin for pathogenic bacterial species, *M. haemolytica* and *P. multocida*. These pathogens are often involved in the complex etiology of a multi-pathogen respiratory disease – BRD. The pharmacodynamic differences of these magnitude, if confirmed by *in vivo* investigations, may require a targeted dosage regimen for different strains of the pathogens^5^. This study contributes to the developing body of literature investigating the potential variation in the antimicrobial PD against bacterial pathogen isolates for which the drug MICs are similar^10,11,16^. Modifications of the antimicrobial treatment regimen depending on the phenotypic properties of the pathogen strain causing disease – *e.g*., its PD responses and growth rate – may be an approach for precision medicine. The types of the modifications needed will require further investigation.

## Material and Methods

### Bacterial isolates

Four isolates of *M. haemolytica* and three isolates of *P. multocida* were obtained from the lungs of cattle affected with BRD under field conditions (the samples were routine submissions to the Kansas State Veterinary Diagnostic Laboratory during 2015–2016). All the isolates of *P. multocida* had typical colony morphology (greyish, spherical, and viscous colonies) on tryptic soy agar supplemented with 5% sheep blood (BAP, Remel^TM^, Lenexa, KS, USA). All the isolates of *M. haemolytica* had typical colony morphology (greyish, spherical, and smooth colonies) on BAP. The species differentiation was confirmed using the indole test and MALDI-TOF mass spectrometry. The bacteria were stored in tryptic soy broth (Remel^TM^, Lenexa, KS, USA) supplemented with 15% glycerol at −80 °C.

### MIC estimation

The minimum inhibitory concentration (MIC) of enrofloxacin for each isolate was first determined by the broth microdilution assay following the standard procedures^22^ in cation-adjusted Mueller-Hinton broth (MHB, Remel^TM^), aerobically, with an inoculum bacterial concentration of ~5 × 10^5^ colony forming units (CFU)/mL^23^. Enrofloxacin was purchased from Sigma-Aldrich, Inc (St. Louis, MO, USA). The quality control strains used in the microdilution assay were *Escherichia coli* ATCC® 25922™ and *Staphylococcus aureus* subsp. *aureus* ATCC® 29213™^24^. The microdilution assay following the standard procedures is based on serial two-fold dilution of the tested antimicrobial concentration^22^; thus, the assay only identifies the double-dilution interval in which the MIC value lies^25^. Because of this, the enrofloxacin MIC estimation for the isolate was further refined by the E-test® performed in accordance with the manufacturer’s recommendations (bioMérieux, Marcy-l'Étoile, France).

### Time-kill experiments

Time-kill experiments with enrofloxacin were conducted with each of the seven bacterial isolates, using previously described procedures^10,12,22^. Specifically, for each isolate, an aliquot of the frozen stock was plated on BAP and incubated aerobically at 37 °C. From the overnight plate, several bacterial colonies were inoculated into brain heart infusion broth (BHI, Remel^TM^), which was incubated aerobically at 37 °C with shaking (200 rpm) overnight. The overnight culture was diluted 1:200 into fresh BHI and incubated at 37 °C for 30 minutes - at which time the bacterial population was growing exponentially - and then diluted 1:20 into each of the individual flasks containing fresh BHI with different concentrations of enrofloxacin (0.5, 0.75, 1, 2, 3, 5, or 10 fold the MIC of the isolate) and into a control flask with fresh BHI (no enrofloxacin added). The starting bacterial concentrations are plotted in Figs 1 and 2. (Note that a fluoroquinolone MIC for a *P. multocida* or *M. haemolytica* isolate remains the same between MHB and BHI^10^; BHI is used for the time-kill experiments^10,12^). The drug-free control and drug-exposure cultures were incubated aerobically with shaking (200 rpm) at 37 °C, and sampled at 0, 1, 2, 3, 4, 5, 6, 7, and 8 hour of the incubation. Previous studies in our laboratory with these bacterial species and enrofloxacin have showed that the initial linear phase of the bacterial population growth/decline is completed within 8 hours of the experiment^12^ (this is also similar to the action of the fluoroquinolone marbofloxacin against these bacterial species^11^). Thus, the *E*_*max*_ model can be fitted to the data from this initial experimental phase^12^. Each sample of the cultures underwent 10-fold serial dilutions in a 0.9% sterile saline solution, and two of the dilutions – chosen based on the expected bacterial concentration – were direct plated on BAP. These plates were incubated aerobically at 37 °C for 24 hours, at which time the colony counts were determined. The counts from the two dilutions of the sample were averaged providing an estimate of the bacterial concentration in the culture (CFU/mL) at the time point.

For each isolate, a time-kill experiment on a given date was performed including the isolate’s control culture (no enrofloxacin added) and its cultures with several of the enrofloxacin concentrations. Five to eight such time-kills experiments were performed with each isolate. This allowed producing the replicates of time-kill data for the isolate for each drug concentration (that is, each concentration 0.5, 0.75, 1, 2, 3, 5, or 10 fold the MIC was tested against the isolate in at least two independent experiments performed on different days), as well as sufficient data for fitting the PD model with acceptable precision of the parameter estimates (see below).

### Pharmacodynamic modeling

For each individual time-kill experiment with the isolate on a given date, for each drug concentration or the control, the determined log_10_-transformed bacterial concentration (log_10_(CFU/mL)) was plotted *vs*. time (*i.e*. on the semi-logarithmic scale). A linear regression model was fitted to the data and the slope provided an estimate of the rate of growth/decline of the bacterial population at that drug concentration or in the control. The growth/decline rate estimates across all drug concentrations and experiments for that isolate were pooled into a new dataset, keeping track of the experiment date. The sigmoid *E*_*max*_ model (Eq. 1) was fitted to the pooled dataset to describe the relationship between the drug concentration and the rate of growth/decline of the bacterial population. For each isolate, the model was fitted to the pooled dataset as a nonlinear mixed-effect regression model using the maximum likelihood method in the Phoenix® NLME software (Certara USA, Inc., Princeton, NJ, USA). The bacterial population growth/decline rate was the outcome, the antimicrobial drug concentration was the fixed effect, and the date of the experiment was the random effect in the model. The model’s goodness of fit to the data for the isolate was evaluated considering the Akaike’s Information Criterion (AIC); visual inspection of the plot of the predicted *vs*. observed rate of the bacterial population growth/decline at different drug concentrations; and visual inspection of the residual diagnostic plots for outliers or trends which would suggest a poor model fit. Once the best-fit PD model form was determined, precision of estimation of the value of each parameter in the model was considered acceptable if the coefficient of variation was ≤ 40%. For each isolate, the time-kill data were amassed until the model parameters were estimated with that precision.

### Availability of data and materials

The experimental data (results of the bacterial time-kill experiments) are included in Figs 1 and 2.
